# ATF3 prevents retinal ganglion cell apoptosis and mitigates microglia-mediated neuroinflammation in retinal ischemia–reperfusion injury

**DOI:** 10.3389/fimmu.2025.1671204

**Published:** 2025-10-16

**Authors:** Ningzhi Zhang, Tian Tian, Zhiyi Wang, Xuejun He, Wenye Cao, Wenxi Zhang, Yiqiao Xing, Ning Yang

**Affiliations:** ^1^ Department of Ophthalmology, Renmin Hospital of Wuhan University, Wuhan, China; ^2^ Department of Ophthalmology, Sir Run Run Shaw Hospital, Zhejiang University School of Medicine, Hangzhou, China; ^3^ Department of Ophthalmology, The First Affiliated Hospital of Yangtze University, Jingzhou, Hubei, China; ^4^ Aier Eye Hospital of Wuhan University, Wuhan, Hubei, China

**Keywords:** retinal ischemia-reperfusion, ATF3, neuroinflammation, retinal ganglion cell, microglia, glaucoma

## Abstract

**Introduction:**

Neuroinflammation is a key pathological response involved in secondary optic nerve injury following retinal ischemia–reperfusion injury. The expression of activating transcription factor 3 (ATF3), a highly conserved protein, is rapidly induced post-injury and is crucial for regulating immunity and inflammation. The potential neuroprotective mechanisms, function, and therapeutic potential of ATF3 following retinal ischemia–reperfusion remain largely unexplored. In this study, we examined the expression and distribution of ATF3 and achieved the overexpression of ATF3 in mouse retina via injection of adeno-associated virus vectors.

**Methods:**

Retinal ganglion cell survival was assessed using immunofluorescence staining and terminal deoxynucleotidyl transferase dUTP nick-end labeling assay. Activation status and polarization of microglia and microglia-associated neuroinflammation were also evaluated. In addition, peripheral venous blood samples and aqueous humor were collected from 20 individuals, 10 patients with primary angle-closure glaucoma and 10 controls, to detect changes in ATF3 expression.

**Results:**

ATF3 overexpression partially suppressed retinal ganglion cell apoptosis by activating the p-Akt pathway, inhibited microglial activation, reversed microglial M1/M2 polarization, and reduced the release of inflammatory factors by decreasing integrin CD11b expression. ATF3 overexpression improved retinal structure and function by regulating microglial behavior and decreased neuronal death post-retinal ischemia–reperfusion.

**Discussion:**

ATF3 overexpression may be a potential therapeutic strategy for the management of retinal ischemia–reperfusion-associated neurodegenerative diseases.

## 1 Introduction

Retinal ischemia–reperfusion (RIR) injury is associated with ocular diseases such as glaucoma and diabetic retinopathy. Progressive retinal ganglion cell (RGC) death triggered by RIR leads to irreversible visual degradation and blindness. In addition to impairing blood flow during the reperfusion phase, ischemia–reperfusion (IR) causes retinal damage due to the generation of inflammatory mediators and free radicals (1). However, knowledge gaps regarding post-IR neuronal death and its underlying mechanisms persist. Inflammatory response is a key pathological process in neurological dysfunction and progressive optic nerve injury. Neuroprotection can be achieved by inhibiting neuroinflammatory responses to alleviate secondary injury (2, 3). Microglia play an important role in innate immunity. They are the first responders to central nervous system (CNS) injury, monitoring their surroundings to maintain homeostasis through scavenging, phagocytosis, and providing neurotrophic support (4). Microglia-related neuroinflammation substantially contributes to RGC loss (5, 6). Under pathological conditions, microglia are activated to a detrimental phenotype, promoting tissue damage or recovery. Initially, microglia enhance their phagocytic capacity to repair tissue damage. However, persistent disease triggers the chronic activation of microglia, which consume both cell debris and healthy photoreceptors. The regulation of microglia-mediated neuroinflammation has gained considerable attention for the repair of CNS injury.

Given the central role of neuroinflammation in propagating secondary damage following RIR injury, targeting key regulatory molecules that modulate this response represents a promising therapeutic strategy. Members of the Activating Transcription Factor (ATF) family play crucial roles as transcriptional regulators in cellular stress responses, metabolism, inflammation, and apoptosis, and have been strongly implicated in various inflammatory disease models and neurodegenerative disorders (7). Among them, ATF3 has garnered significant attention in recent years. As a key stress-inducible regulator, ATF3 is involved in cellular adaptation to diverse insults, including ischemic injury. In contrast, pathological stimuli, including axotomy, IR, and neurotoxicity, upregulate ATF3 expression. ATF3 can confer robust neuroprotective effects and potentially promote axonal regeneration post-injury (8–11). ATF3 is a conserved pro-regenerative factor across vertebrates (10). ATF3 overexpression before ONC promotes RBPMS^+^ RGC survival and preserves RGC function (8). In early developing cortical neuronal cells of opossum, inhibition of ATF3 expression decreases neurite outgrowth and differentiation and increases cell death, indicating the significance of ATF3 in CNS development (12). Furthermore, ATF3 expression in the CNS is closely related to microglial activation. The ATF3/c-Jun/Lgals3 axis may regulate microglial activation state and phagocytic ability after hypothalamic pituitary stalk injury (13). ATF3 represses neuronal apoptosis and cerebral ischemia-induced microglial activation via CCL2 targeting (14). Furthermore, in stressed microglia, ATF3 expression is considerably up-regulated, which may contribute to the transcriptional repression of interferon-regulated genes, regulating stress-induced neuroinflammation (15).

Evidence suggests that ATF3 may exert conserved protective effects in RIR injury across various organs, such as heart, kidney and brain, primarily through suppression of inflammatory pathways and regulation of stress-related molecules (16–19). This provides important mechanistic insight for further investigation into its role in RIR injury. However, the potential neuroprotective mechanisms of ATF3 post-RIR remain elusive. Therefore, here, we aimed to determine the role of ATF3 in neuronal degeneration and microglial behavior post-RIR and elucidate the underlying mechanism.

## 2 Materials and methods

### 2.1 Animals

C57BL/6J male mice (6–8 weeks of age), procured from the Laboratory Animal Center of Wuhan University, were housed in cages with free access to food and water and were subjected to a 12-/12-h dark/light cycle. Animal experiments were performed in accordance with the ARVO Statement for the Use of Animals in Ophthalmic and Vision Research and approved by the Laboratory Animal Ethics Committee of the Renmin Hospital of Wuhan University.

### 2.2 Intravitreal injection

Recombinant adeno-associated virus vector serotype 2 (rAAV2) was purchased from Brain VTA (Wuhan, China) and prepared according to standard procedures. The open reading frame (ORF) of ATF3 (ID: NM_007498.3, 545bp) was cloned and inserted into the vector. The other elements in the vector were as follows: rAAV-EF1a-ATF3-P2A-EGFP-WPRE-hGH polyA (vector from Brain VTA; titer 5.45E + 12 μg/mL). Corresponding empty vectors (rAAV-EF1a-EGFP-WPRE-hGH polyA, titer 5.60E + 12 μg/mL) were used as negative controls. Four weeks before establishing the experimental glaucoma model, a unilateral intravitreal injection containing 1 μL of AAVs was administered into the right eye of the mice. Before injection, the mice were anaesthetized with an intraperitoneal injection of 2% pentobarbital sodium at a dose of 40-50 mg/kg and topical oxybuprocaine hydrochloride eye drops to minimize discomfort.

### 2.3 RIR injury induction

Mice were anaesthetized with an intraperitoneal injection of 2% pentobarbital sodium at a dose of 40-50 mg/kg. Mydriasis was induced using topical tropicamide ophthalmic solution (Sinqi Pharmaceutical Co., Ltd., China). The anterior chambers (ACs) were cannulated using a 32G needle attached to a normal saline reservoir, elevated to 150 cm, and maintained for 60 min. This procedure maintained an average intraocular pressure (IOP) of 80 mmHg. The IOP immediately returned to normal levels following needle withdrawal. The contralateral eye with rapid puncture of the AC without elevated IOP served as a control. The retinas were collected 1-, 3-, and 7-days post-surgery for subsequent experiments. We established the following three groups: CON (control mice with sham operation), EGFP+IR (mice sampled 3 days after RIR, injected the control virus vector 4 weeks prior to sampling), and ATF3oe+IR (mice sampled 3 days after RIR, injected the rAAV-ATF3-EGFP vector 4 weeks prior to sampling) (n=3-6 per group). Eyes with cannulation-induced cataracts, iris injury, iris bleeding, or AC leakage were excluded.

### 2.4 RGC quantification

The eyes of the mice were extracted and fixed in 4% paraformaldehyde for 45 min to perform retinal flat mounting. The retinas were blocked with 5% bovine serum albumin (BSA) and 0.5% Triton X-100 in phosphate-buffered saline (PBS) and incubated with a Brn3a antibody (**Supplementary Table S3**). After gentle rinsing in PBS (thrice), the retinas were incubated with Alexa Fluor 594 (Jackson ImmunoResearch Laboratories). Subsequently, the retinas were subjected to four radial incisions and flattened on a glass slide to form a ‘petal’ shape. A fluorescence microscope (BX51; Olympus, Tokyo, Japan) was used to photograph the retinal flat mounts. Three fields from each petal were selected at radial distances of 1/6, 1/2, and 5/6 from the optic nerve head, resulting in 12 fields. Surviving RGCs were counted using ImageJ software (National Institutes of Health, USA).

### 2.5 Immunofluorescence staining of retinal microglia

The frozen retinal tissue sections (14 μm) were washed with PBS with Tween^®^ detergent (PBST) at room temperature and blocked in bovine serum albumin and 0.3% Triton X-100 (BSAT) for 1 h, and incubated overnight with primary antibodies (Iba-1) at 4 °C. Following incubation, the samples were incubated with species-specific secondary fluorescent antibodies for 1 h. The nuclei were stained with DAPI. Retinal flat mounts were prepared as described previously. Subsequently, the retinas were incubated with primary antibodies (Iba1, CD16/32, and CD206; **Supplementary Table S3**) for 72 h at 4 °C, and then with secondary antibodies for 2 h at room temperature. The following secondary antibodies were used: Alexa Fluor 647 IgG (#ANT034; AntGene Biotechnology), Alexa Fluor 594 IgG (#711-585-152; Jackson ImmunoResearch Laboratories), and Alexa Fluor 405 IgG (#ANT038; AntGene Biotechnology). The retinal flat mounts were photographed using a laser scanning confocal microscope (FV1200; Olympus, Tokyo, Japan). The z-stack of the scanning range began when the microglia first became visible and ended when they were no longer visible, with a series of images obtained every 1 μm. The images were overlaid, and microglia throughout the retina were counted using ImageJ. Microglial morphology was analyzed as described previously (20).

### 2.6 Hematoxylin and eosin staining

Following euthanasia by cervical dislocation, the mouse eyes were enucleated at designated time points, fixed with FAS (Servicebio), and embedded in paraffin. Subsequently, the eyeballs were dehydrated with an alcohol gradient and embedded in wax. Three sections of thickness 4 μm were obtained across the optic nerve of each eye and stained with hematoxylin and eosin (HE). The sections were observed under an Olympus microscope (Olympus, Germany). Images were quantitatively analyzed using ImageJ, and eight evenly spaced points of each section were selected for measurement. Cell nuclei rows of retinal ONL were manually quantified at 800 µm from the center of the optic disc. The average of three sections per eye was taken (n=5 per group).

### 2.7 Terminal deoxynucleotidyl transferase dUTP nick end labeling assay

Terminal deoxynucleotidyl transferase dUTP nick end labeling (TUNEL) of 14-μm-thick retinal tissues was performed to evaluate apoptosis (21). The cryosections were incubated with TUNEL detecting solution (Beyotime Biotechnology, Shanghai, China) at 37 °C for 1 h in the dark. After washing three times in PBS for 10 min each, the sections were labeled with DAPI to stain cell nuclei. Finally, the retinal sections were analyzed using confocal laser scanning microscopy.

### 2.8 RNA isolation, reverse transcription, and reverse transcription-quantitative poly-merase chain reaction

Total RNA was extracted from the retina and reverse-transcribed to cDNA using the reverse transcription kit (R222-01; Vazyme Biotech Co., Ltd., Nanjing, China) per the manufacturer’s instructions. SYBR Green polymerase chain reaction (PCR) (Q711-02; Vazyme Biotech Co., Ltd.) was performed using a BIO-RAD 7500 PCR system. The sequences of primers used are presented in **Supplementary Table S2**. The reaction conditions were as follows: stage 1, 95 °C for 30 s; stage 2, 95 °C for 10 s; stage 3, 60 °C for 30 s; repeated 40 times; stage 4, 95 °C for 15 s; stage 5, 60 °C for 1 min; stage 6, 95 °C for 15 s. Relative gene expression was calculated using the 2−ΔΔCT method. All experiments were performed in triplicate.

### 2.9 Protein extraction and western blotting

Following eye removal, the retinas were quickly isolated on ice and transferred into RIPA lysis solution containing phenylmethanesulfonyl fluoride and a protease inhibitor cocktail. After complete tissue lysis, the supernatant was collected using centrifugation after homogenization. Protein concentrations were measured using a bicinchoninic acid protein assay kit (EpiZyme, Shanghai, China) per the manufacturer’s instructions. Proteins (30 μg) were separated using 10% sodium dodecyl sulfate-polyacrylamide gel electrophoresis and transferred onto a polyvinylidene fluoride membrane. The membrane was blocked with 5% skimmed milk in Tris-buffered saline solution containing 0.2% Tween-20 and incubated with the primary antibodies listed in **Supplementary Table S3**. GAPDH was used as a housekeeping protein. After incubation with horseradish peroxidase (HRP)-labeled goat anti-rabbit IgG (Servicebio, Wuhan, China) and anti-HRP mouse monoclonal antibody (Servicebio), labeled proteins were developed using enhanced chemiluminescence reagents, and bands were analyzed using ImageJ.

### 2.10 Electroretinogram analysis

The mice were anaesthetized with an intraperitoneal injection of 2% pentobarbital sodium at a dose of 40-50 mg/kg and had their pupils dilated with 1% tropicamide in a dark environment after an overnight dark adaption. Two gold electrodes on the ocular surface, a reference electrode needle inserted subcutaneously between the ears, and a ground electrode in the tail were used to measure the signals. For dark-adapted electroretinography (ERG), white flashes of 0.001, 0.003, 0.010, 0.030, 0.100, 0.300, 1.000, 3.000, and 10.000 cd·s/m2, with 1-min intervals between flashes, were delivered using the RetiMINER-C visual electrophysiological system (IRC Medical Equipment Co., Ltd., Chongqing, China). Measurements of a- and b-wave amplitudes at 0.030, 0.100, 0.300, 1.000, and 3.000 cd·s/m2 were recorded. The oscillatory potentials (OPs) were measured at a flash stimulus intensity of 3.0 cd·s/m2, and the sum of the three main wavelet amplitudes was recorded. After dark adapted ERG measurement, the mice were exposed to bright adaptation background light for 10 min and light adaptation was detected at a flash stimulus intensity of 10.0 cd·s/m2, and the amplitude of a- and b-waves was recorded.

### 2.11 Clinical sample collection

Ten patients diagnosed with primary APACG who received surgical treatment in the Ophthalmology Department of Jingzhou First People’s Hospital of Hubei Province from November 2024 to December 2024 were included. Ten patients with simple senile cataract who were admitted for surgical treatment during the same period comprised the CON group. All included patients underwent comprehensive and standardized medical history collection and eye examination.

The specific inclusion criteria included the following: (1) age range: 40–80 years; (2) patients presented with the first attack in one eye and within 1 week of the APACG; (3) an acute increase in IOP with corresponding ocular and systemic clinical symptoms; and (4) the angle of the diseased eye can be observed under the atrial angle microscope. The specific inclusion criteria for patients with senile cataract included the following: (1) age range: 40–80 years; (2) the diagnosis of senile cataract and met the standards for senile cataract surgery; and (3) the angle of the room was open under the angle mirror.

The exclusion criteria for all patients were (1) secondary glaucoma and special types of glaucoma, such as malignant tumor secondary glaucoma, malignant glaucoma, neovascular glaucoma, and uveal inflammatory glaucoma; (2) cataract caused by other factors such as non-age-related factors; (3) cataract in the dilatation stage or hypermaturity stage; (4) received eye surgery or laser treatment; (5) combined with other eye diseases, such as corneal ulcer, endophthalmitis, retinitis pigmentosa, and retinal detachment; (6) suffering from serious systemic diseases, such as thyroid-related eye diseases, autoimmune diseases, serious cardiovascular and cerebrovascular liver and kidney diseases, and infectious diseases; and (7) unable to cooperate with examination or treatment, such as patients with cognitive impairment or mental illness.

In addition to blood collection for routine preoperative examination, 2 mL of fasting venous blood was collected and placed in dry vacuum sampling vessels without anti-coagulants. Carefully collected the serum, marked with patient information, and stored in an ultra-low temperature refrigerator at −80 °C for later use. Aqueous humor (80–100 μL) was collected following standard aseptic procedure, while the patients’ affected eyes were subjected to appropriate surgical treatment. All surgical procedures were performed by senior physicians.

### 2.12 Enzyme-linked immunosorbent assay

A human ATF3 enzyme-linked immunosorbent assay (ELISA) test kit (Bioswamp Life Science Lab, Wuhan, China) was used per the manufacturer’s instructions.

### 2.13 Statistical analysis

Data are presented as mean ± standard error of the mean. One-way analysis of variance was performed to compare data among multiple groups, followed by Tukey’s honest significant difference test. t-test was performed to compare two groups of independent measurement data when the data conformed to normal distribution and homogeneity of variance. The Fisher’s exact test was used for binary traits (sex, diabetes, and hypertension). All statistical analyses were conducted using GraphPad Prism 8.0.2 (GraphPad, San Diego, CA, USA). Results with P < 0.05 were considered significant.

## 3 Results

### 3.1 Retinal expression of ATF3 increases post-RIR injury

**Figure 1:**
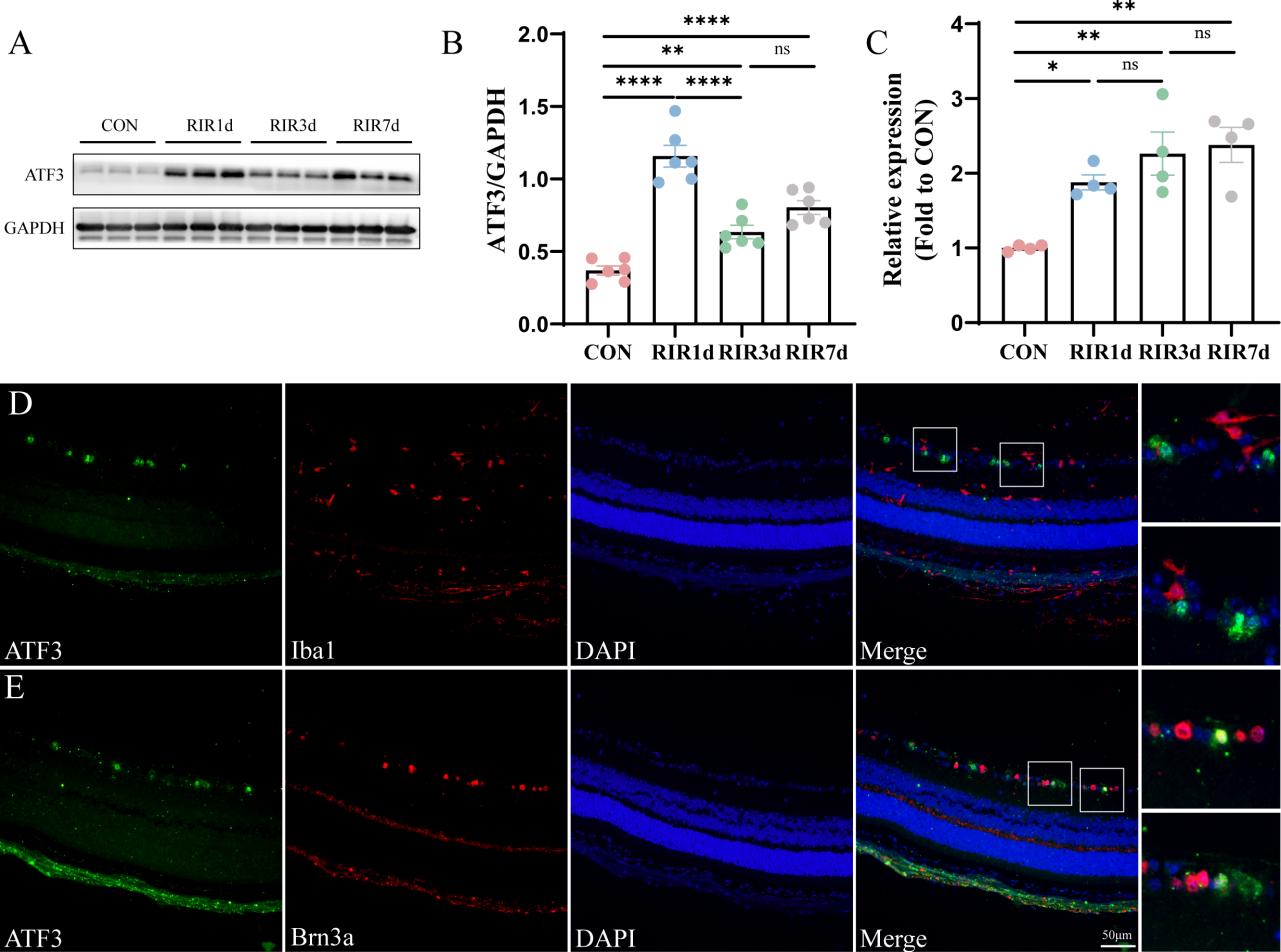
Time course and spatial ATF3 expression post-retinal ischemia–reperfusion injury. **(A, B)** Western blot representing retinal ATF3 expression (n = 6, **P < 0.01, ****P < 0.0001, ns, not significant). Data are expressed as mean ± standard error of the mean. **(C)** qRT-PCR analysis of retinal ATF3 mRNA expression (n = 4, *P < 0.05, **P < 0.01, ns, not significant). **(D, E)** Immunofluorescence images of retinal sections stained with ATF3 (green), Brn3a or Iba1 (red), and DAPI (blue) in the RIR 3-day group. CON, control; RIR, retinal ischemia–reperfusion.

Western blotting revealed that ATF3 expression increased significantly on day 1 post-RIR and subsequently decreased; however, on days 3 and 7, ATF3 expression in the RIR group was significantly higher than that in the CON group (**Figures 1A, B**). ATF3 transcription continued to increase for 7 days post-RIR injury (**Figure 1C**). RGC proportion in the retina of mice was approximately 87% on day 1 (comparable with that in the CON group), 64% on day 3, and 46% on day 7 (**Supplementary Figure S1**). Three days after RIR was selected as the follow-up study time point. Double immunofluorescence staining was performed to detect the cellular localization of ATF3 at 3 days post-RIR. ATF3 was mainly expressed in the ganglion cell layer (GCL) after RIR injury and partially colocalized with the RGC nucleus (**Figures 1D, E**).

### 3.2 ATF3 overexpression improves retinal structure

**Figure 2:**
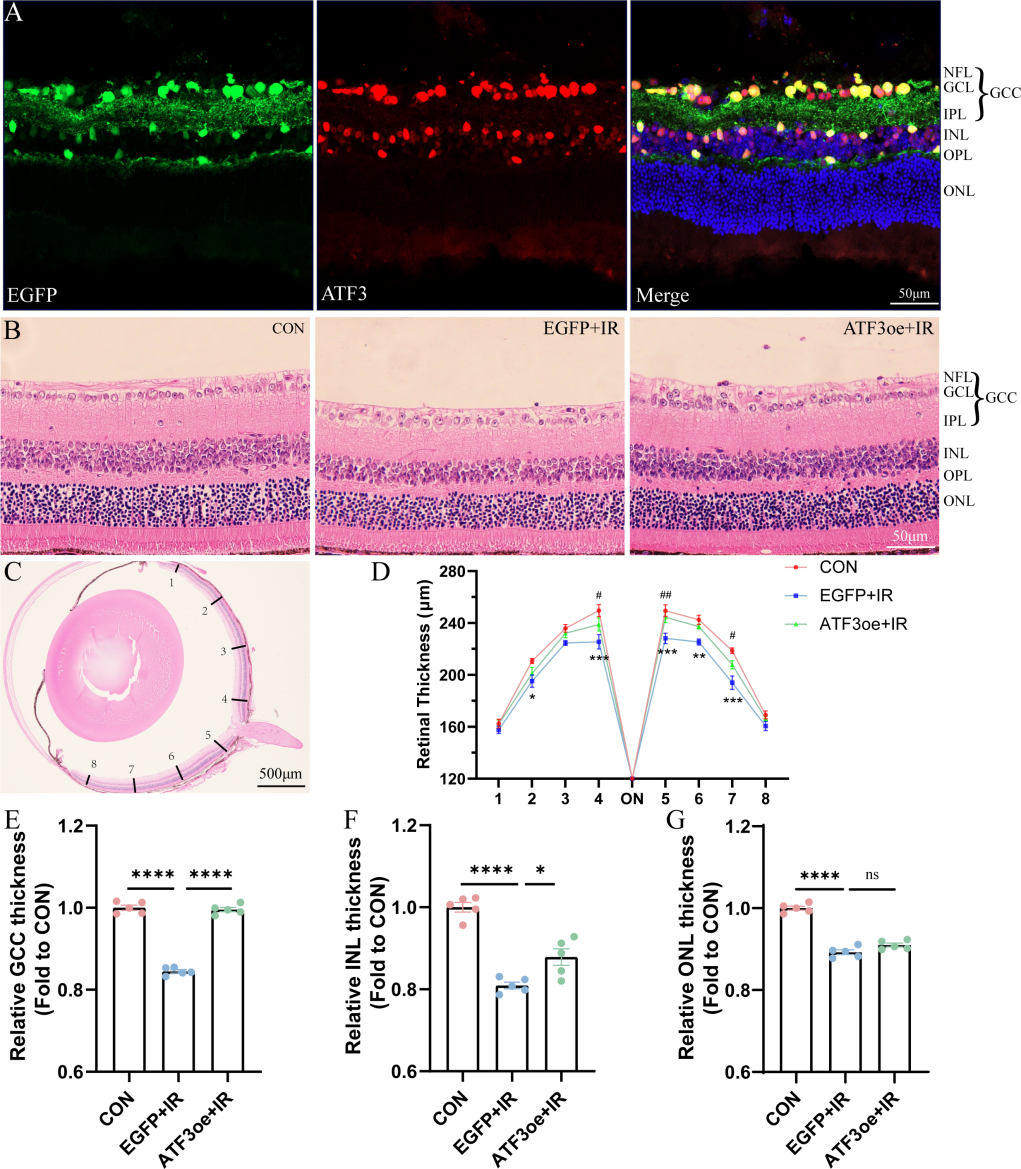
Effects of ATF3 overexpression on retinal structure. **(A)** Immunofluorescence images of retinal sections stained with EGFP (green), ATF3 (red), and DAPI (blue) after intravitreal injection of rAAV-ATF3-EGFP. **(B, C)** Representative images of hematoxylin-eosin-stained retinal sections. **(D-G)** Statistical analysis of thickness of the entire retina, GCC, INL, and ONL. *P values in blue are representative of the EGFP+IR versus CON groups. #P values in green are representative of the ATF3oe+IR versus EGFP+IR groups. GCC, ganglion cell complex; INL, inner nuclear layer; ONL, outer nuclear layer. Data are expressed as mean ± standard error of the mean. n = 5, *P < 0.05, **P < 0.01, ***P < 0.001, ****P < 0.0001, ns, not significant. ^#^P<0.05, ^##^P<0.01.

We constructed an ATF3-overexpressing AAV vector and successfully overexpressed ATF3 in GCL, inner nuclear layer (INL), and, outer plexiform layer (OPL) of the mouse retina (**Figure 2A**; **Supplementary Figure S2**). We administered an intravitreal injection of rAAV-ATF3-EGFP or rAAV-EGFP 4 weeks before AC perfusion. HE staining was used to measure and compare the thicknesses of the entire retina, retinal GCC, INL, and outer nuclear layer (ONL) (**Figure 2B**). Three days post-IR injury, we observed a significant decline in the thickness of the entire retina in the EGFP+IR group, and ATF3 overexpression could preserve retinal GCC and INL thickness, while exerting no significant effect on the thickness of the ONL (**Figures 2C–G**). We further quantified the number of nuclear rows in the ONL. In the CON group, 13.33 ± 0.65 layers of ONL nuclei were present, in the EGFP+RIR group, 10.67 ± 0.68 layers of ONL nuclei were observed; and in the ATF3oe+RIR group, 11.73 ± 0.19 layers of ONL nuclei were detected. Similarly, there was no statistically significant difference in the number of nuclear layers in the retinal ONL between the EGFP+RIR group and the ATF3oe+RIR group (p= 0.3886, n=5).

### 3.3 ATF3 overexpression improves visual function

**Figure 3:**
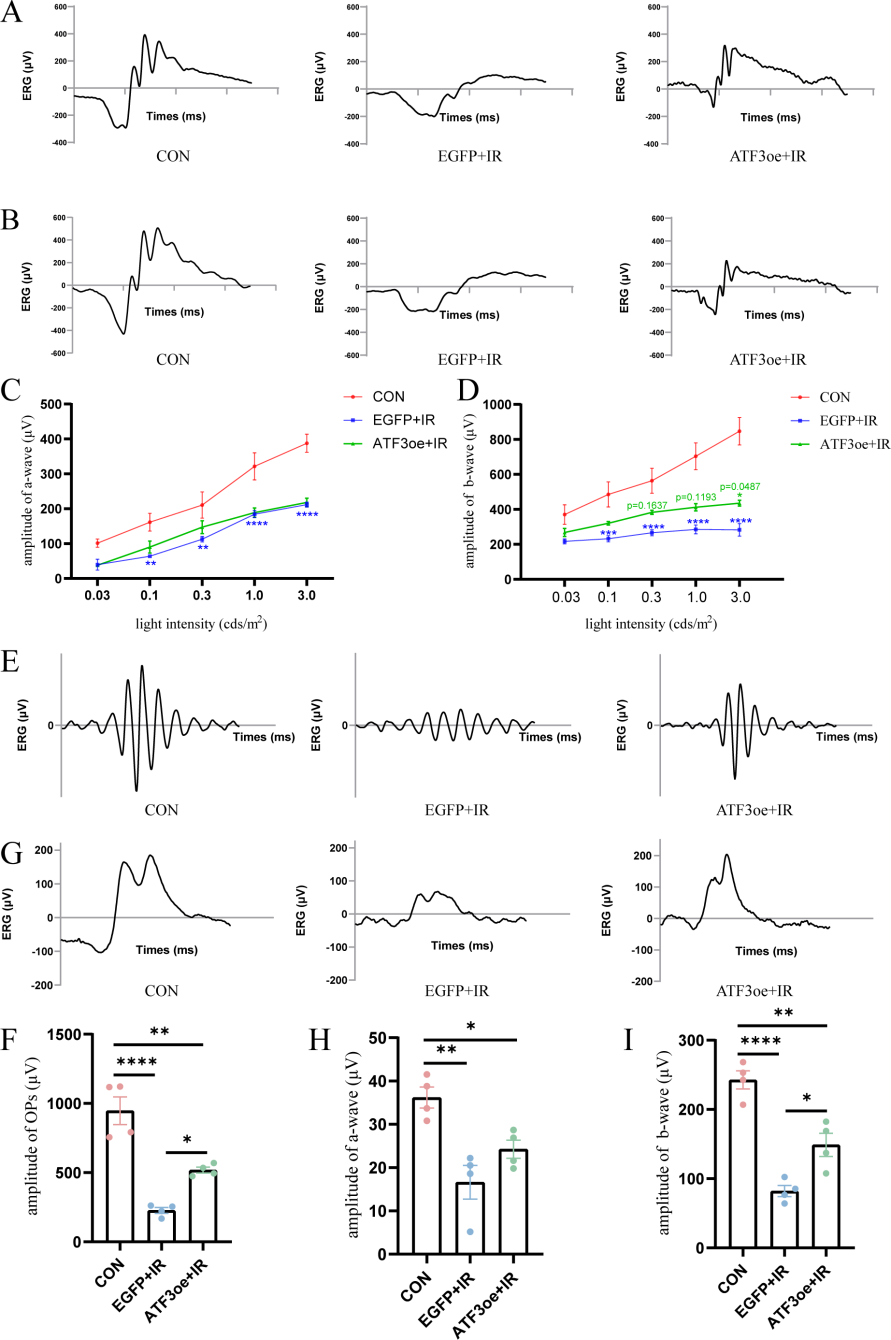
Effects of ATF3 overexpression on retinal function. **(A, B)** Representative images of ERG were recorded at 1.0 and 3.0 cd·s/m2 in the CON, EGFP+IR, and ATF3oe+IR groups. **(C, D)** The bar charts representing the amplitudes of a- and b-waves in the three aforementioned groups at 0.03, 0.1, 0.3, 1.0, and 3.0 cd·s/m2. P values in green are representative of the ATF3oe+IR versus EGFP+IR groups. P values in blue are representative of the EGFP+IR versus CON groups. **(E)** Representative images of dark-adapted OPs recorded at 3.0 cd·s/m^2^ in the CON, EGFP+IR, and ATF3oe+IR groups. **(G)** Representative images of light-adapted ERG under a light stimulus intensity of 10.0 cd·s/m^2^ in the CON, EGFP+IR, and ATF3oe+IR groups. **(F)** Bar chart showing the changes in dark-adapted OPs wave amplitude in mice from each group. **(H, I)** Bar chart showing the changes in light-adapted ERG b-wave amplitude under a light stimulus intensity of 10.0 cd·s/m^2^ in mice from each group. *P<0.05, **P < 0.01, ***P < 0.001, ****P < 0.0001. CON, control; ERG, electroretinogram; IR, ischemia–reperfusion.

The a- and b-waves at 1.0 and 3.0 cd·s/m² became indistinct post-RIR injury (**Figures 3A, B**). Additionally, the amplitudes of the a- and b-waves decreased significantly post-RIR injury, indicating retinal dysfunction. At the flash intensity of 3.0 cd·s/m², we observed a b-wave amplitude of 847.2 ± 78.5 μV in the CON group, 286.6 ± 36.3 μV in the EGFP+IR group, and 435.1 ± 16.6 μV in the ATF3oe+IR group. These results demonstrate that ATF3 overexpression significantly attenuated the RIR-induced reduction in the b-wave amplitude (P = 0.0487). In contrast, no significant differences were observed in the a-wave amplitudes among the groups (**Figures 3C, D**). Dark-adapted OPs were recorded across groups, with total amplitude quantified as the sum of three principal peaks (**Figures 3E, F**). The CON group demonstrated an OP amplitude of 947.2 ± 100.2 μV, which decreased to 226.9 ± 21.3 μV in the EGFP+IR group. ATF3 overexpression significantly attenuated this reduction. The statistical analysis also revealed ATF3 overexpression specifically rescued b-wave amplitudes of light-adapted ERG without significant alteration in the a-wave (**Figures 3G, H, J**).

### 3.4 ATF3 overexpression attenuated neuronal apoptotic death via the p-AKT pathway post-RIR

**Figure 4:**
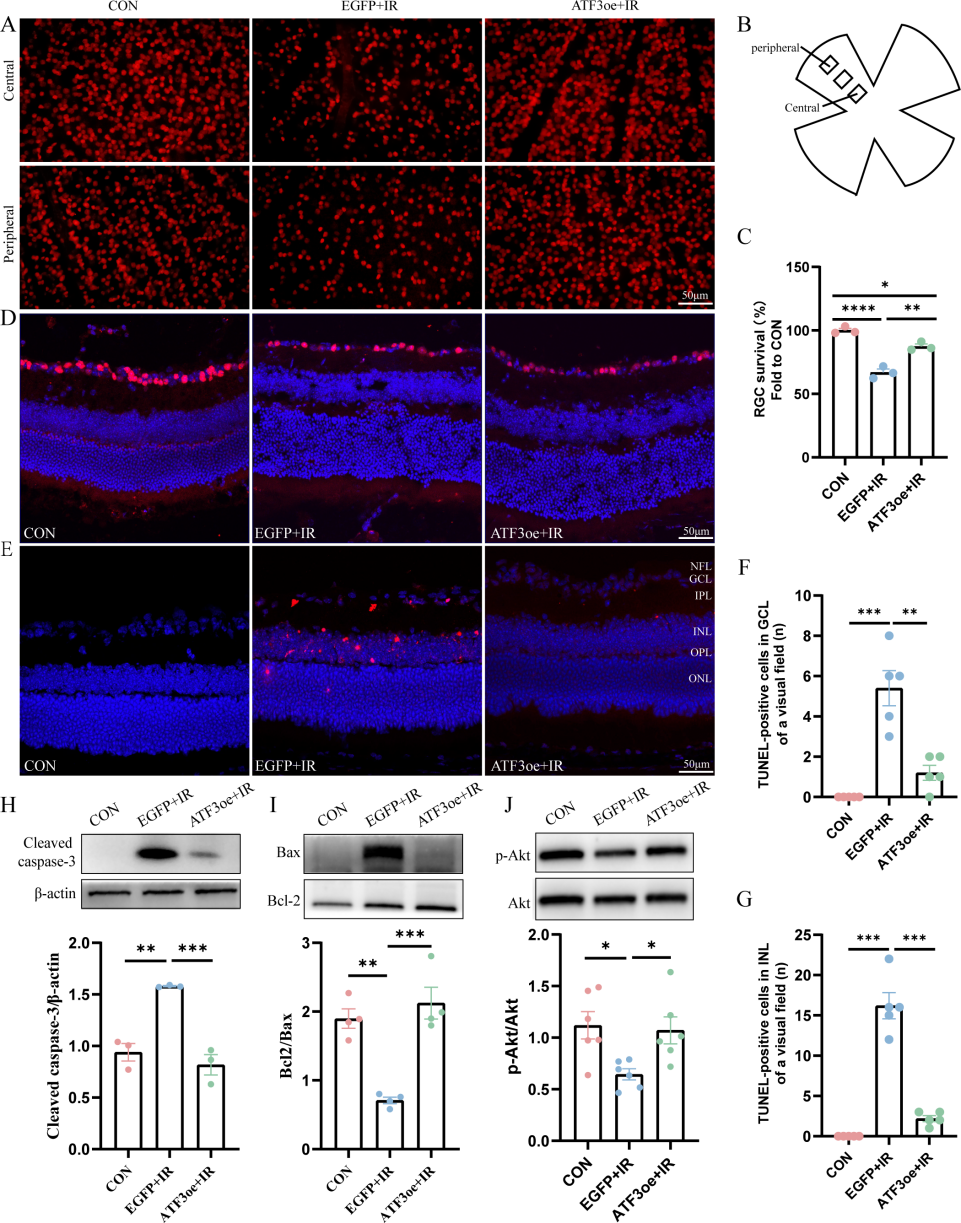
Effects of ATF3 overexpression on RGC apoptosis. **(A)** Immunofluorescence images of retinal flat-mounts stained with Brn3a (red) to visualize RGCs. **(B)** Schematic diagram of field of view selection. **(C)** RGC survival rates in each group (n = 3). **(D)** Immunofluorescence images of retinal sections stained with Brn3a (red) and DAPI (blue). **(E)** Retinal sections were stained with TUNEL (red) and DAPI (blue) to examine apoptotic cells and nuclei. **(F, G)** The number of TUNEL-positive cells in GCL or INL of a visual field. **(H-J)** Western blot analysis of apoptosis-related molecules (cleaved caspase-3, Bax, and Bcl-2) and phospho-Akt/Akt pathway proteins 3 days post-RIR injury. Data are expressed as mean ± standard error of the mean. *P < 0.5, **P < 0.01, ***P < 0.001, ****P < 0.0001, CON, control; RGC, retinal ganglion cell; IR, ischemia–reperfusion.

After a 3-day reperfusion period, RGC count significantly decreased, as evidenced by immunostaining of both whole-mount retinas and frozen retinal sections with Brn3a antibodies. ATF3 overexpression enhanced RGC survival in IR-injured retinas (**Figures 4A–D**). Apoptosis is a common mode of RGC death during the early stages of RIR. TUNEL staining of the retinal sections further confirmed that ATF3 significantly inhibited the apoptosis of retinal GCL and INL cells (**Figures 4E–G**). ATF3 overexpression significantly reduced cleaved caspase-3 level and significantly increased Bcl-2/Bax levels. Addition-ally, the p-Akt/Akt ratio was significantly higher in the ATF3 overexpression group than in the EGFP+IR group, suggesting that ATF3 may inhibit apoptosis post-RIR, partially by up-regulating the p-Akt pathway (**Figures 4H–J**).

### 3.5 ATF3 overexpression prevents microglial overactivation

**Figure 5:**
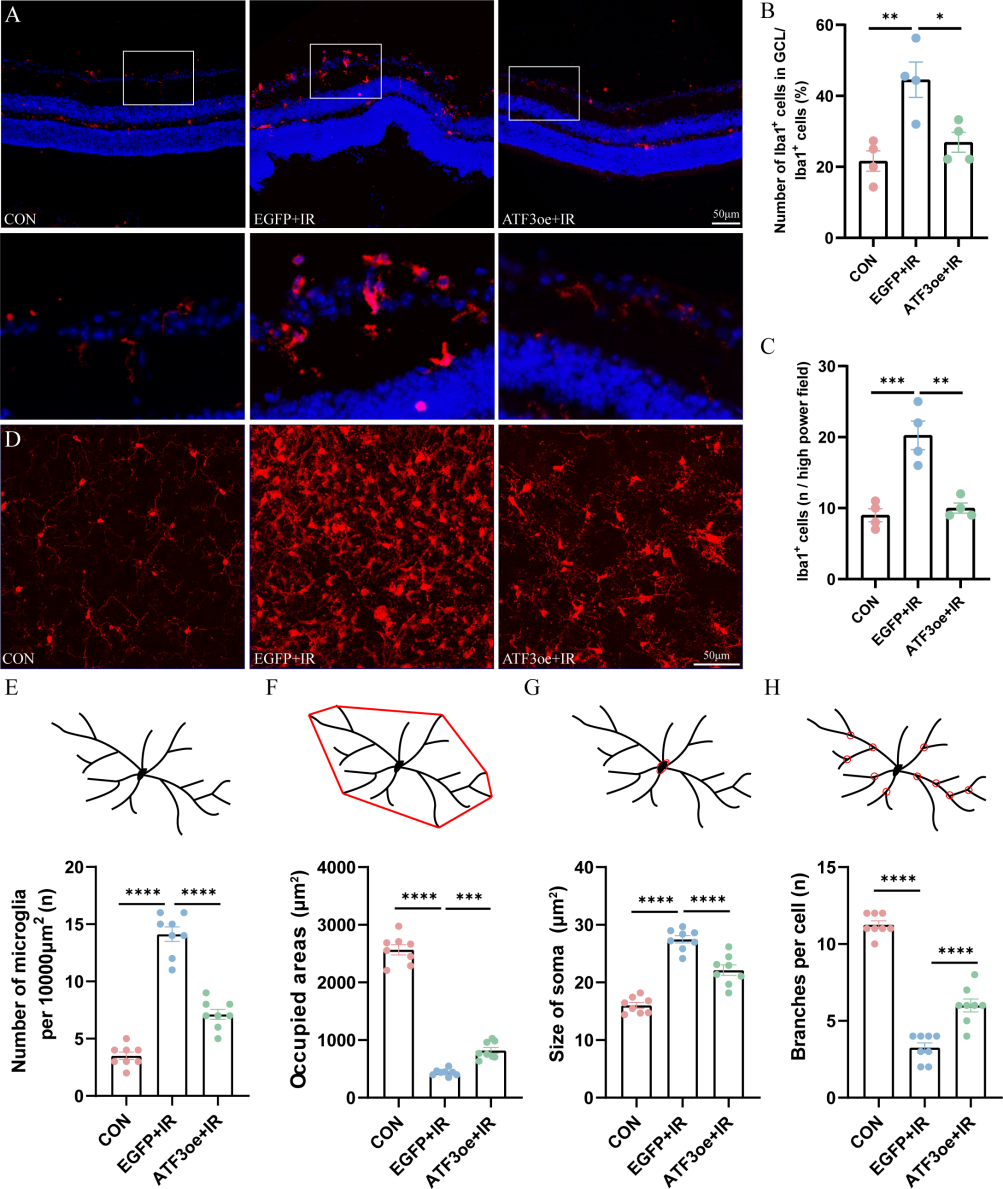
Effects of ATF3 overexpression on microglial activation. **(A)** Microglia stained with iba-1 in retinal sections. **(B)** Proportional changes of microglia localized to the retinal GCL relative to the total retinal microglial population. **(C)** The number of Iba1-positive cells per high power field. **(D)** Microglia stained with iba-1 in retinal flat-mount preparations. **(E–H)** The number of microglia, microglia-occupied area, the size of soma, and the number of process branches per microglia were measured or counted. Results are ex-pressed as mean ± standard error of the mean. *P<0.05, **P<0.01, ***P < 0.001, ****P < 0.0001. CON, control; GCL, ganglion cell layer; IR, ischemia–reperfusion.

We determined whether the overexpression of ATF3 affect the behavior of immune cells. Immunofluorescence staining of the retinal sections revealed that microglia were significantly activated and that there was a proportional increase in Iba1^+^ cells in the GCL relative to the pan-retinal Iba1^+^ cell population post-RIR injury (**Figures 5A–C**). By day 3 post-RIR injury, retinal immunofluorescence staining showed that the number of microglia increased and number of branches decreased, presenting an amoeboid appearance. ATF3 overexpression may indirectly inhibit excessive activation of microglia by reducing RGCs damage. Microglia count in the ATF3 overexpression group was lower than that in the control virus-injected group 3 days post-RIR injury, with a larger cell area, smaller cell body area, and greater number of preserved branches (**Figures 5D–H**). This result suggests that microglia in ATF3-overexpressing retinas are morphologically closer to the inactivated retina.

### 3.6 ATF3 overexpression regulates retinal microglial polarization

**Figure 6:**
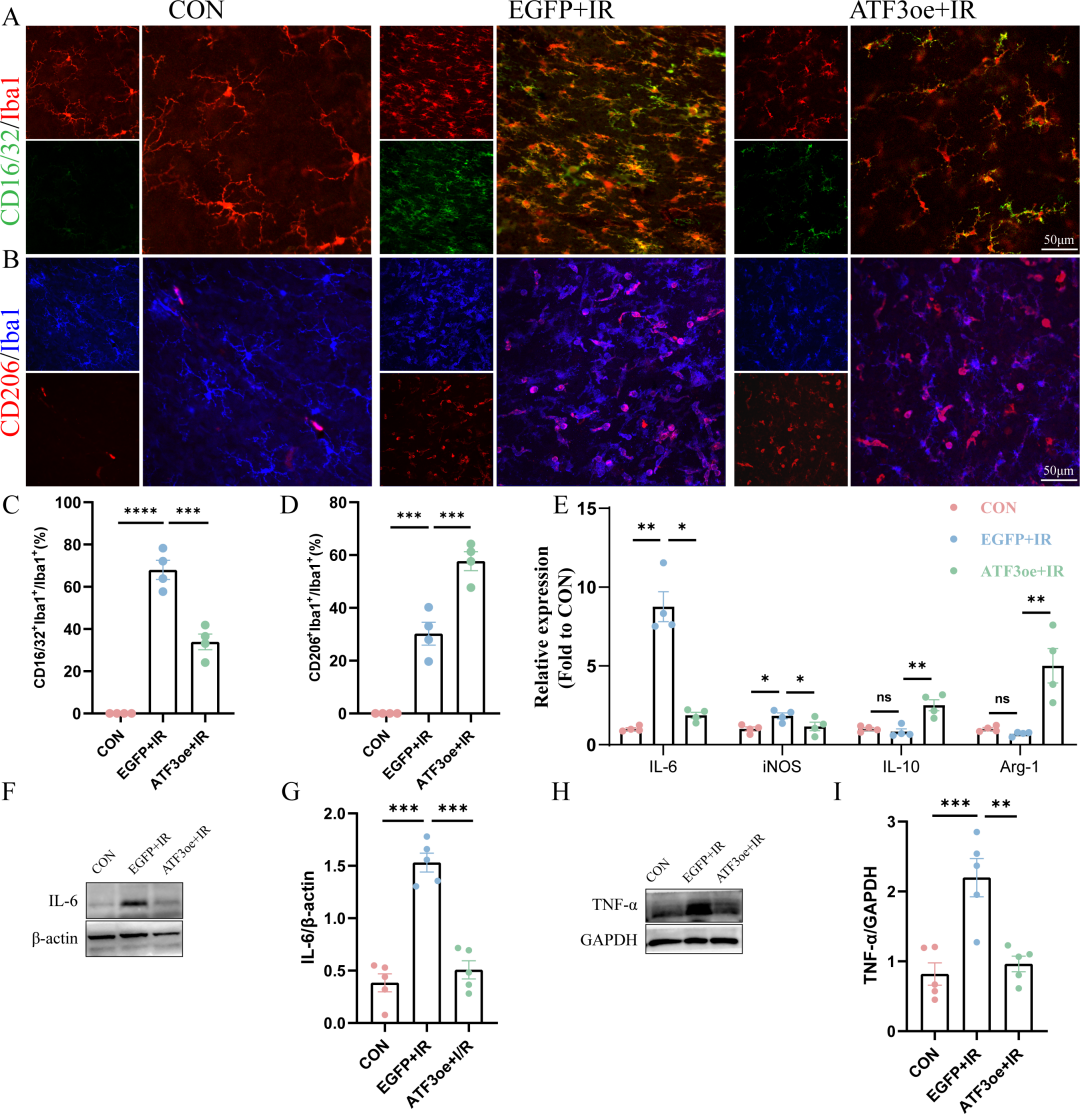
Effects of ATF3 overexpression on microglial polarization and neuroinflammatory response. **(A)** Retinal flat mounts were co-stained with CD16/32 (green) and Iba1 (red) to visualize proinflammatory microglia. Retinal flat mounts were co-stained with CD206 (red) and Iba1 (blue) to examine anti-inflammatory microglia. **(C)** Proportional changes in CD16/32+Iba1+ microglia relative to the total retinal microglial population. **(D)** Proportional changes in CD206+Iba1+ microglia relative to the total retinal microglial population. **(E)** iNOS, Arg-1, IL-6, and IL-10 mRNA levels were determined using real-time qPCR in purified retina. GAPDH was used as the ‘housekeeping’ gene. **(F-I)** Western blot analysis of IL-6 and TNF-α. Data are shown as mean ± standard error of the mean. *P < 0.05, **P < 0.01, ***P < 0.001, ****P < 0.0001. CON, control; IL-6, interleukin-6; IL-10, interleukin-10; iNOS, inducible nitric oxide synthase; IR, ischemia–reperfusion.

In the early stages of RIR injury, retinal microglial cells are activated and polarized into different phenotypes, including the pro-inflammatory M1 and anti-inflammatory M2 phenotypes. We detected microglial subtype markers (M1: FCγRIII/FCγRII (CD16/32), inducible nitric oxide synthase (iNOS), and interleukin (IL)-6; M2: mannose receptor (CD206), arginase-1 (Arg-1), and IL-10) and used immunofluorescence staining and qRT-PCR analysis to investigate the effect of ATF3 overexpression on microglial polarization post-RIR injury. The ratio of M1/M2 increased rapidly in the RIR group after 3 days, and ATF3 overexpression reversed the ratio of M1/M2 post-RIR by inhibiting the M1 phenotype and promoting the M2 phenotype (**Figures 6A–E**). Additionally, ATF3 over-expression suppressed the release of the inflammatory cytokines IL-6 and tumor necrosis factor (TNF)-α (**Figures 6F-I**).

### 3.7 ATF3 overexpression decreases neuronal inflammation partially via inhibition of CD11b expression

**Figure 7:**
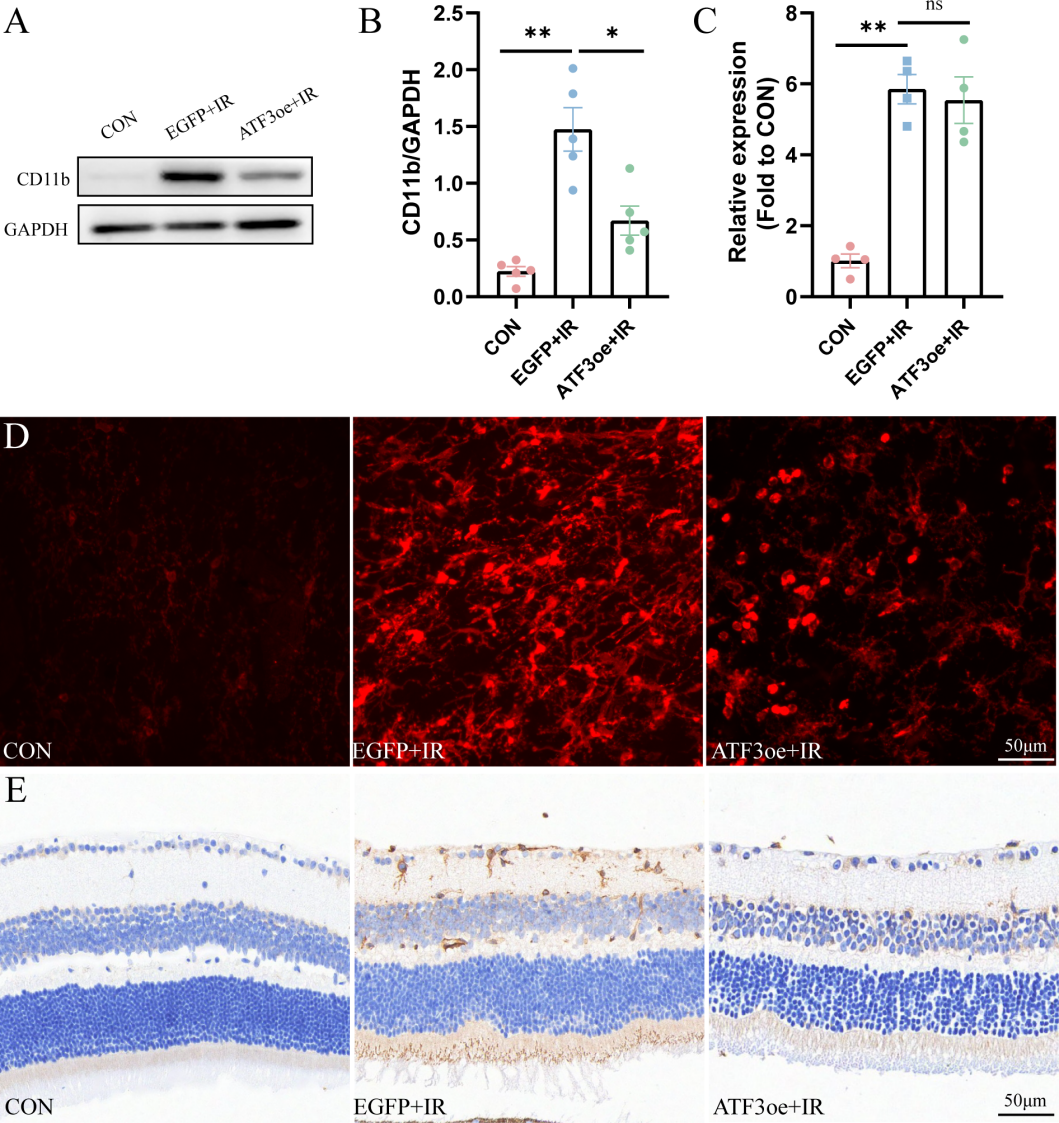
Overexpression of ATF3 decreases the expression of CD11b. **(A, B)** Western blot analysis showing that ATF3 overexpression reduces CD11b expression. **(C)** qRT-PCR analysis of CD11b. **(D)** Multilayer overlay image of retinal flat-mounts stained with antibody against CD11b (red). **(E)** Retinal sections are stained with antibody against CD11b. Data are shown as mean ± standard error of the mean. *P < 0.05, **P < 0.01, ns, not significant.

CD11b is a member of the adhesion receptor beta 2 integrin family, expressed on most immune cells. We observed that CD11b expression in the retina of mice post-RIR injury was significantly up-regulated compared with that in the CON group, and ATF3 overexpression could significantly inhibit the increase in CD11b expression post-RIR injury (**Figures 7A, B**). The qRT-PCR analysis showed that ATF3 overexpression had no significant effect on CD11b mRNA levels (**Figure 7C**). The confocal scanning overlay of CD11b immunofluorescence staining showed that the number of CD11b^+^ cells increased significantly post-RIR modeling, and ATF3 overexpression could effectively alleviate the phenomenon (**Figure 7D**). The CD11b immunohistochemical results of retinal sections of mice in each group also confirmed these findings (**Figure 7E**).

### 3.8 ATF3 expression in the serum and aqueous humor of patients with APACG and controls

**Figure 8:**
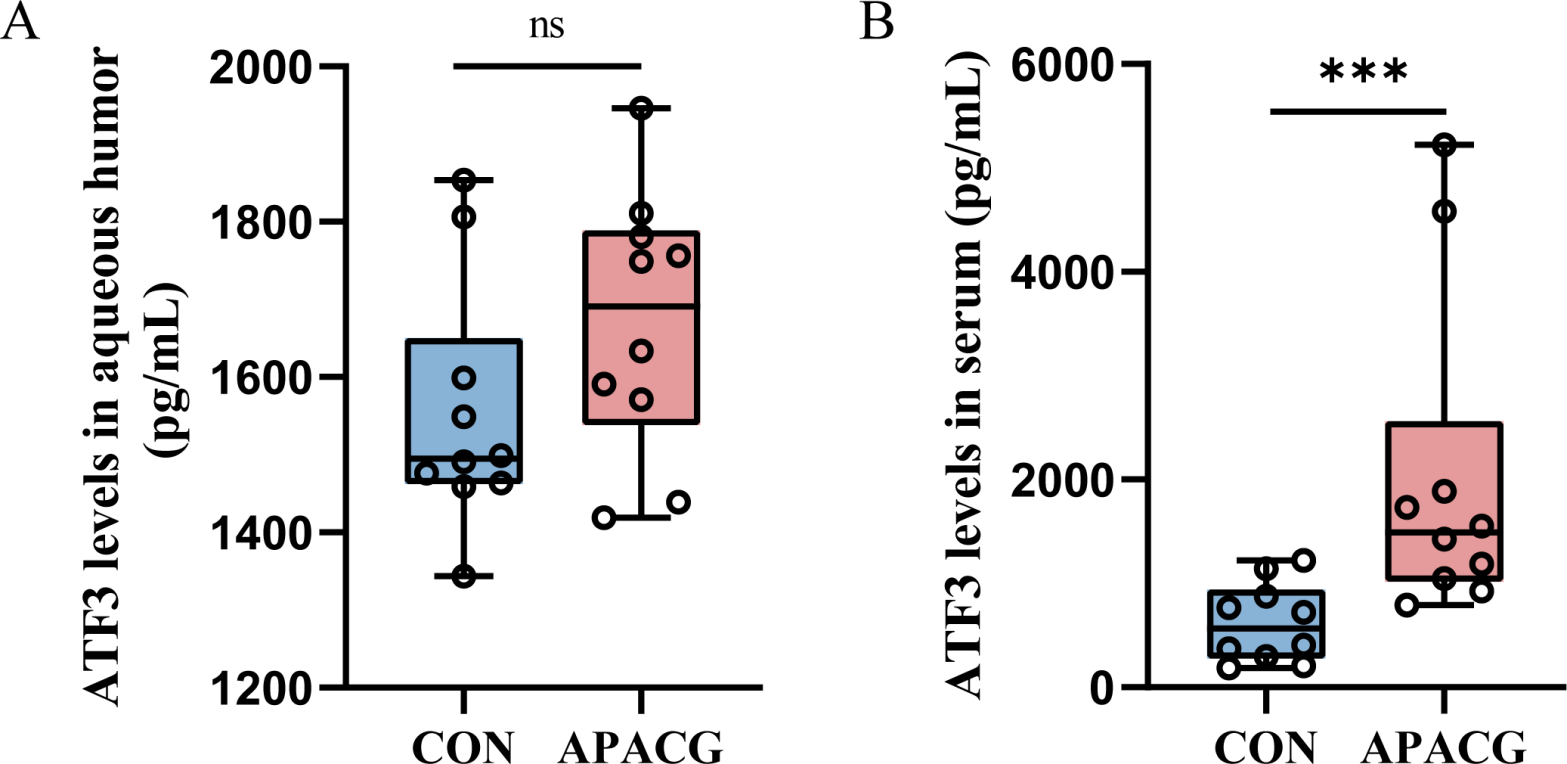
ATF3 expression in patients with PACG and controls. **(A)** Aqueous humor; **(B)** serum. Data are shown as median and quartiles, n = 10, ***P < 0.001, ns, not significant.

As patients were matched for age, sex, body mass index, history of systemic hypertension, and diabetes, no significant differences were found in these characteristics between the groups (**Supplementary Table S1**). In the APACG cohort, the visual field parameters were as follows: mean deviation = 6.72 ± 0.69 dB; pattern standard deviation = -13.72 ± 1.80 dB. There was no significant difference in the expression level of ATF3 in aqueous humor between the APAGC and CON group, and ATF3 expression in the serum of patients with APAGC was significantly higher than that in the serum of controls (**Figures 8A, B**).

## 4 Discussion

This study demonstrated that ATF3 overexpression improved short-term retinal structure and visual function, suppressed neuronal death, and inhibited microglial overactivation. Moreover, up-regulation of ATF3 expression was associated with down-regulation of TNF-α and IL-6 expression. ATF3 may potentially attenuate RGC apoptosis and thereby inhibit microglia-induced neuroinflammation by regulating the p-Akt and integrin CD11b signaling pathways.

ATF3 expression is up-regulated in a wide range of pathological conditions. For instance, ATF3 expression is substantially up-regulated in both mouse and rat models of brain ischemic and reperfusion injuries (22). In central retinal artery ligation (CRAL)-induced retinal ischemia, ATF3 was reportedly activated 30 min post-CRAL and 3 h post-reperfusion (23). ATF3 may be one of the core transcriptional factors involved in RGC degeneration after ONC (24). Here, ATF3 expression sharply increased and peaked 1 day post-RIR, and this is consistent with the previous study findings. In a study that identified the top up-regulated common genes in IOP rats, ATF3 expression was up-regulated by 3.24- and 28.38-fold in the entire retina and RGCL, respectively (25). Here, we found that ATF3 expression on day 3 post-RIR was mainly localized to the GCL and that it may be closely related to RGC degeneration.

A previous study reported that on day 3 post-RIR injury in mice, the retinal IPL thickness decreased significantly (26), consistent with the observations of this study. Another study using HE staining and SD-OCT scanning showed that, compared with the control eye, RIR eye showed thickening due to edema in the early stage and then continued thinning accompanied by cell loss; the IPL and INL showed the greatest reduction in thickness (27). Neurofilaments are essential for the structural integrity of axons, and a study has revealed that ischemic optic nerve neurofilament structure distortion and myelin destruction could be observed 21 days post-RIR injury in rats (28). Another study reported neurofilament abnormalities in the optic nerve 3 days after modeling in an experimental glaucoma model of high IOP induced by laser photocoagulation (5). The number and distribution of axons and myelin of optic nerve can be better observed using toluidine blue staining in semithin section of optic nerve.

ATF3 plays a protective role in CNS injuries. In a previous study, ATF3-knockout mice, compared to wild-type mice, exhibited worse neurological outcomes and large damaged regions after SCI or ischemic stroke, indicating that ATF3 has a neuroprotective function (29). ATF3-knockout mice exhibit larger infarcts and worse functional outcomes after middle cerebral artery occlusion (30). Here, as expected, we found that ATF3 overexpression attenuated RIR-induced RGC death. The retinal electrical reaction to a light stimulus is recorded using ERG. The magnitude and temporal pattern of its constituent a-wave, b-wave, and OPs can reflect the retina’s function to some extent. Nevertheless, the amplitudes of the a- and b-waves did not show significant differences with or without ATF3 overexpression 3 days post-RIR injury in this study; however, a protective tendency was observed. The a-wave mainly reflects the activity of rods and cones, and the b-wave mainly reflects the function of ON-bipolar and Müller cells. Whether ATF3 overexpression can affect ON-bipolar or Müller cells is worth further investigation. Larger samples, longer duration of injury, and other waveforms of different cell origins, such as the PhNR, and Pattern ERG are required.

Apoptosis is the key mechanism of cell death during early RIR injury. In the retinal tissues post-IR, severe retinal destruction is initiated, inflammatory factor expression is increased, and apoptosis is up-regulated. Caspase-3, which is critical for modulating the apoptotic pathway, is a common marker of both pathways. The combination of ATF3 and c-Jun induces the expression of the antiapoptotic factor Hsp27, which activates Akt directly or indirectly, potentially inhibiting apoptosis and inducing neurite outgrowth (31). Additionally, ATF3 can inhibit the expression of cerebral ischemic injury-mediated apoptosis carboxy-terminal modulatory protein, a pro-apoptotic factor that inhibits the anti-apoptotic Akt/PKB cascade (32). Through apoptosis-related molecule detection and tunnel assays, we found that ATF3 overexpression reduced apoptosis post-RIR. IR induces various types of programmed cell deaths. One research group found increased expression of many genes that regulate apoptosis, necroptosis, pyroptosis, ferroptosis, and parthanatosis in RGCs isolated from the retinas 24 h after IR (33). The combination of apoptosis, necrotic apoptosis, and ferroptosis inhibitors is more effective in preventing IR-induced RGC death than any inhibitor alone (34). Therefore, therapies that concurrently regulate the activity of multiple PCD pathways and reduce RGC death post-IR are required.

Microglia are activated within minutes after the start of an ischemia episode, which causes the release of several proinflammatory substances and exacerbates brain damage (35, 36). Following ischemia, microglial proliferation peaks at 48–72 h and persists for a few weeks after the initial injury (37, 38). Neuroinflammation occurs in the early stages of RIR injury. Additionally, here, we found that the RGC count decreased most rapidly from days 1 to 3 post-RIR injury, with a slightly flatter rate from days 3 to 7. Therefore, we selected day 3 post-RIR as the time point to explore whether up-regulated ATF3 expression could maintain the homeostasis of microglia and inhibiting microglia-mediated neuroinflammation by protecting RGCs during the acute phase of RIR. Microglia in the CNS are involved in various functions such as defense, tissue repair, and immunoregulation (39). Exploring the transformation mechanism between different microglial phenotypes is important for the balance of the brain immune system. A previous study revealed that ATF3 triggers M2 macrophage polarization to protect against inflammatory injury during sepsis via the ILF3/NEAT1 axis (40). ATF3 reverses M1 polarized macrophages to the M2 phenotype by up-regulating tenascin expression via the Wnt/β catenin signaling pathway (41). We found that ATF3 overexpression inhibited the abnormal activation of microglia in the early stages of RIR injury and promoted their polarization toward the M2 phenotype. Its specific mechanism needs to be further elucidated. Recently, single-cell sequencing and spatial transcriptomics have been used to comprehensively analyze the molecular characteristics of microglia, revealing more subtypes, providing more precise cell targets for future basic research and clinical treatment (42). The simple M1/M2 dichotomy after stimulation is insufficient to classify microglia subpopulations, and several *in vivo* studies of M1/M2 microglia may have mistaken infiltrating macrophages for the classically defined ‘M2’ phenotype microglia. Currently, the use of CD16/32^+^Iba1^+^ cells to represent the M1 phenotype and CD206^+^Iba1^+^ cells to represent the M2 phenotype is widely adopted in the field (43–45). Our results showed that ATF3 overexpression reduces the proportion of CD16/32^+^Iba1^+^ cells in the retina of mice 3 days post-RIR injury, while increasing the proportion of CD206^+^Iba1^+^ cells. Future studies need to further refine the differentiation between retinal microglia and infiltrating macrophages. To understand the roles of ATF3 in the nervous system comprehensively, more advanced conditional knockouts in neurons, glial cells, and other immune system cells should be established (46). Future studies should clarify the indirect or direct association between ATF3 overexpression and changes in microglia. Supplementary temporal analysis should be conducted to determine the sequence of events between changes in microglia and the protective effect on RGCs; alternatively, direct tests could be performed in microglial cell lines (e.g., BV2 cells) to observe whether ATF3 overexpression can prevent activation induced by stressors such as lipopolysaccharide.

CD11b is encoded by Itgam and constitutes the α chain of integrin Mac-1 (also known as CD11b/CD18 or CR3), which is highly expressed in innate immune cells such as monocytes/macrophages and microglia (47). Itgam, as one of the potential core therapeutic targets in patients with ischemic stroke, may play a role by regulating the secretion of key inflammatory factors, IL-1β, IL-6 and TNF-α, and influencing neuronal apoptosis (48). CD11b has been shown to regulate the activation status and function of microglia in stimulation-mediated neuroinflammation and neurodegeneration (49, 50). In neuroinflammatory diseases, gene elimination of fibrinogen binding motif recognized by the microglial integrin receptor CD11b/CD18 can inhibit perivascular micro-glial aggregation and axonal injury (51). Functional ablation of CD11b can reduce β-glucan-induced ocular inflammation in mice, protect the blood–retinal barrier, and enhance RGC axonal regeneration after optic nerve crush injury (52). Studies have revealed the key role of CD11b in balancing pro-inflammatory and anti-inflammatory responses during the disease process. The results of this study show that ATF3 over-expression can down-regulate CD11b expression. Combined with existing reports, the decreased expression of CD11b may be associated with the activation of microglia and regulation of neuroinflammation, and ATF3 overexpression plays a role in neuroinflammation inhibition, at least partially, by down-regulating CD11b expression. ATF3 overexpression had no significant effect on the mRNA level of CD11b, suggesting that ATF3 does not play a role by directly or indirectly regulating the transcription of CD11b, but may affect the post-transcriptional modification or post-translational degradation of CD11b, and the specific regulatory mechanism needs to be explored further. Contrary to our conclusion, one study reported that CD11b deficiency can intensify retinal microglia activation and increase pro-inflammatory cytokine release after acute optic nerve injury (53). The difference can be attributed to the fact that CD11b-knockout mice were directly used in the present study, and the inhibition mechanisms of CD11b overexpression were different between the studies. Additionally, the animal models used were different, namely, optic nerve crush injury and RIR injury models. These differences may lead to variations in the downstream pathways that CD11b activates or inhibits. As a member of the integrin family, the functional role of CD11b is highly cell-type and context-dependent. In acute optic nerve injury models, CD11b deficiency may impair the phagocytic clearance capacity of microglia, leading to the accumulation of damage-associated molecules and subsequently exacerbating microglial activation and pro-inflammatory cytokine release. In contrast, in our RIR model, ATF3 overexpression-induced downregulation of CD11b likely contributes to neuro-inhibition primarily by reducing the migration and adhesion of microglia to the injury site and/or modulating their polarization. Furthermore, CD11b signaling engages multiple downstream pathways, including PI3K/Akt and NF-κB. The strength and nature of the injury stimulus (e.g., mechanical crush vs. ischemic-hypoxic insult) in different models may determine the preferential activation of pro-inflammatory or anti-inflammatory pathways, ultimately leading to distinct functional outcomes.

A study revealed that in patients with silent cerebral infarction or ischemic stroke, the serum ATF3 level elevated within 24 h (29). This phenomenon reveals the potential of ATF3 as a minimally invasive marker of CNS injury. Early symptoms of glaucoma are not obvious, and early detection and diagnosis can be challenging owing to poor clinical efficacy of the available methods and markers. Therefore, understanding the changes in ATF3 expression in the aqueous humor or serum of RIR mice and the blood of patients with different degrees of glaucoma will reveal whether ATF3 can be employed as a clinical diagnostic or prognostic marker for optic neurodegenerative diseases. This study revealed significant up-regulation of ATF3 expression in peripheral blood serum samples from patients with glaucoma. ATF3 is not a recognized secreted protein, and its expression detected in serum may be attributed to the burst release of dead cells; the specific mechanism needs further investigation. More patients with different types of glaucoma and glaucoma at different stages should be included in future studies, in order to further explore the potential of ATF3 as a clinical marker of glaucoma.

There are some limitations to our study. First, we used Brn3a to label RGCs, which has qualitative and quantitative advantages over RBPMS in terms of the reliability of immunohistochemical labeling and manual counting of RGCs (54). However, Brn3a is expressed in only about 80% of RGCs. In rats and mice, Brn3A is expressed in vision-forming RGCs but not in m^+^ RGCs (55). To further study the changes of different subtypes of RGCs, selecting pan-RGC markers is necessary. Second, only 3 days post-RIR was selected as the main time point for exploration. ATF3 overexpression may provide a new therapeutic intervention for ischemia-mediated retinal injury. Whether the expression of recombinant ATF3 can play a protective role over a shorter period requires further investigation. Additionally, the study time point should be extended to explore whether ATF3 can continue exerting a protective role and to determine the degree of protection upon extending the duration of injury. Beyond that, in this experiment, only male mice were selected as research subjects to eliminate the potential interference of gender differences on the experimental results. Considering that gender factors may affect the expression pattern and functional effects of ATF3 by regulating *in vivo* hormone levels, metabolic pathways, and signal transduction networks, etc., it is of great research value and application significance to further explore the differences in the role of ATF3 overexpression in mice of different genders in future studies.

In this study, we chose preventive treatment to investigate the effect of ATF3 on RIR model establishment. Although preventive treatment can explore the feasibility and potential mechanism of gene therapy to a certain extent, therapeutic treatment is more consistent with clinical practical applications. Notably, confirming that “supplementing ATF3 after injury still works” serves as a critical foundation for the subsequent development of ATF3-related clinical treatment protocols. Thus, we need to further verify this in subsequent studies—for example, by injecting ATF3-mimetic peptides into the vitreous cavity of mice after model establishment to verify the clinical translation value of ATF3 overexpression. Currently, viral vectors remain a challenge for clinical translation and are generally not accepted as first-line therapies. Intravitreal drug injection is still the preferred option for the treatment of retinal diseases in clinical practice. With the rapid development of nanocarriers, in the future, we can consider encapsulating ATF3-mimetic peptides, mRNA encoding ATF3 protein, or small-molecule drugs that activate ATF3 expression into nanocarriers (such as polymeric NPs) for delivery to specific lesioned tissues or cells. This approach can combine the advantages of targeted delivery and sustained release (56).

In conclusion, ATF3 overexpression can reduce the apoptosis of retinal ganglion cells (RGCs) via the pAkt/Akt pathway, a process that may further promote the maintenance of microglial homeostasis and alleviate microglia-mediated neuroinflammation; in turn, the alleviated neuroinflammation can further protect RGCs from damage. ATF3 may be involved in the inflammatory communication between neurons and microglia during retinal ischemia. Moreover, ATF3 is a potential peripheral blood marker for the early diagnosis of PACG.

## Data Availability

The original contributions presented in the study are included in the article/**Supplementary Material**. Further inquiries can be directed to the corresponding authors.
