# Supplementary material for: ATF3 prevents retinal ganglion cell apoptosis and mitigates microglia-mediated neuroinflammation in retinal ischemia–reperfusion injury
Source: Front Immunol. 2025 Oct 16;16:1671204. doi: 10.3389/fimmu.2025.1671204 (PMC12571733; doi:10.3389/fimmu.2025.1671204)
Supplement: Supplementary file 1 [file DataSheet1.pdf]

Supplementary Material

Supplementary Tables

Supplementary Table 1. Comparison of Baseline Clinical Characteristics Between Groups

Parameter	CON group (*n* = 10)	PACG group (*n* = 10)	*P*-values
Age (years)	62.00 ± 2.675	65.00 ± 2.936	0.4599[a]
Gender (male/female)	5 (50%)/5 (50%)	4 (40%)/6 (60%)	> 0.9999[b]
Laterality (left/right)	4 (40%)/6 (60%)	5 (50%)/5 (50%)	> 0.9999[b]
Body Mass Index (kg/m^2)	22.40 ± 0.8087	21.60 ± 0.7748	0.4864[a]
Diabetes Mellitus (yes/no)	0 (0%)/10 (100%)	0 (0%)/10 (100%)	> 0.9999[b]
Hypertension (yes/no)	2 (20%)/8 (80%)	1 (10%)/9 (90%)	> 0.9999[b]
Intraocular Pressure (yes/no)	13.70 ± 0.5972	51.50 ± 2.548	< 0.0001[a]

Superscript 'a' denotes *P*-values obtained by independent samples *t*-test, while 'b' indicates *P*-values calculated using Fisher's exact test.

Supplementary Table 2. Primer sequences used for qPCR amplification

Target Genes	Forward primers (5’-3’)	Reverse primers (5’-3’)
IL-6	GCCTTCTTGGGACTGATGCT	TGCCATTGCACAACTCTTTTC
iNOS	GGAGATGGTCCGCAAGAGAG	TCCTGAACGTAGACCTTGGG
IL-10	GCTCTTACTGACTGGCATGAG	CGCAGCTCTAGGAGCATGTG
Arg-1	GTCAGTGTGGTGCTGGGTGG	TGGTTGTCAGGGGAGTGTTG
Atf3	ACCGTCAACAACAGACCCC	CCAGTTTCTCTGACTCTTTCTGC
CD11b	CTTCTGGTCACAGCCCTAGC	GGGGGACAGTAGAAACAGCC
Gapdh	TCGCTCCTGGAAGATGGTGAT	CAGTGGCAAAGTGGAGATTGTTG

Supplementary Table 3. Primary antibodies used in this study

Antibody	Source	Catalog No.	Dilution
Iba1	Wako	WTH2055	IF (1:500)
CD16/32	BD Pharmingen	553141	IF (1:200)
CD206	R&D Systems	AF2535	IF (1:50)
CD11b	CST	17800S	WB (1:1,000); IF (1:500)
Brn3a	Synaptic Systems	411003	IF (1:500)

Antibody	Source	Catalog No.	Dilution
Bax	HuaBio	ET1603-34	WB (1:5,000)
Bcl-2	HuaBio	ET1702-53	WB (1:5,000)
Cleaved-caspase3	CST	9664S	WB (1:1,000)
p-AKT	CST	4060S	WB (1:1,000)
AKT	CST	4691S	WB (1:1,000)
p38 MAPK	CST	8690S	WB (1:1,000)
p-p38 MAPK	CST	4511S	WB (1:1,000)
ATF3	CST	18665S	WB (1:1,000); IF (1:200)
TNF-α	HuaBio	HA722022	WB (1:1,000)
IL-6	CST	12912S	WB (1:1,000)
CD3ζ	Santa	sc-1239	WB (1:1,000)
GAPDH	Servicebio	GB11002	WB (1:8,000)
β-tubulin	Servicebio	GB12139	WB (1:5,000)
β-actin	Servicebio	GB15003	WB (1:5,000)

CST, Cell Signaling Technology. IF, immunofluorescence. WB, western blot.

Supplementary Figures

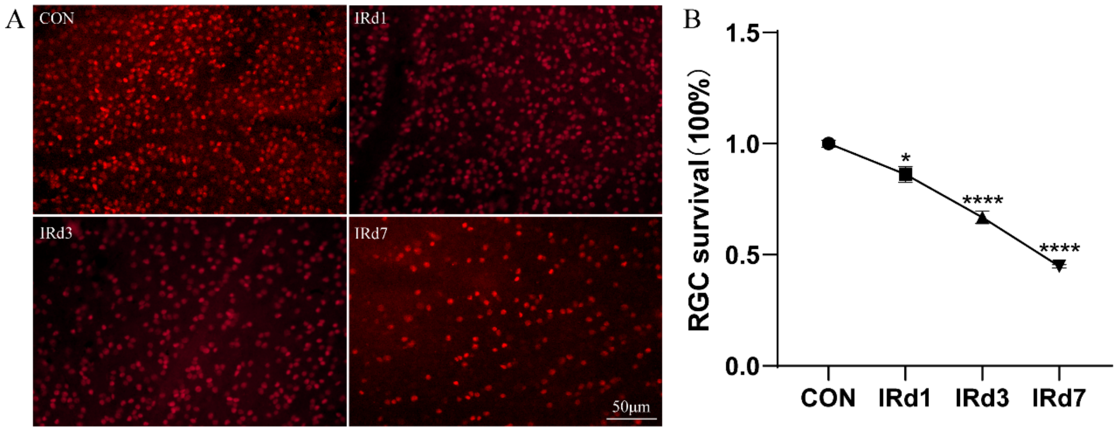

Supplementary Figure 1. RGC quantity with RIR progression over time. (A) Retinal flat mounts stained with Brn3a (red) to visualize RGCs under a fluorescence microscope. Magnification × 400. Scale bar = 50 μm. (B) Statistic analysis of (A). *$P < 0.05$, ****$P < 0.0001$.

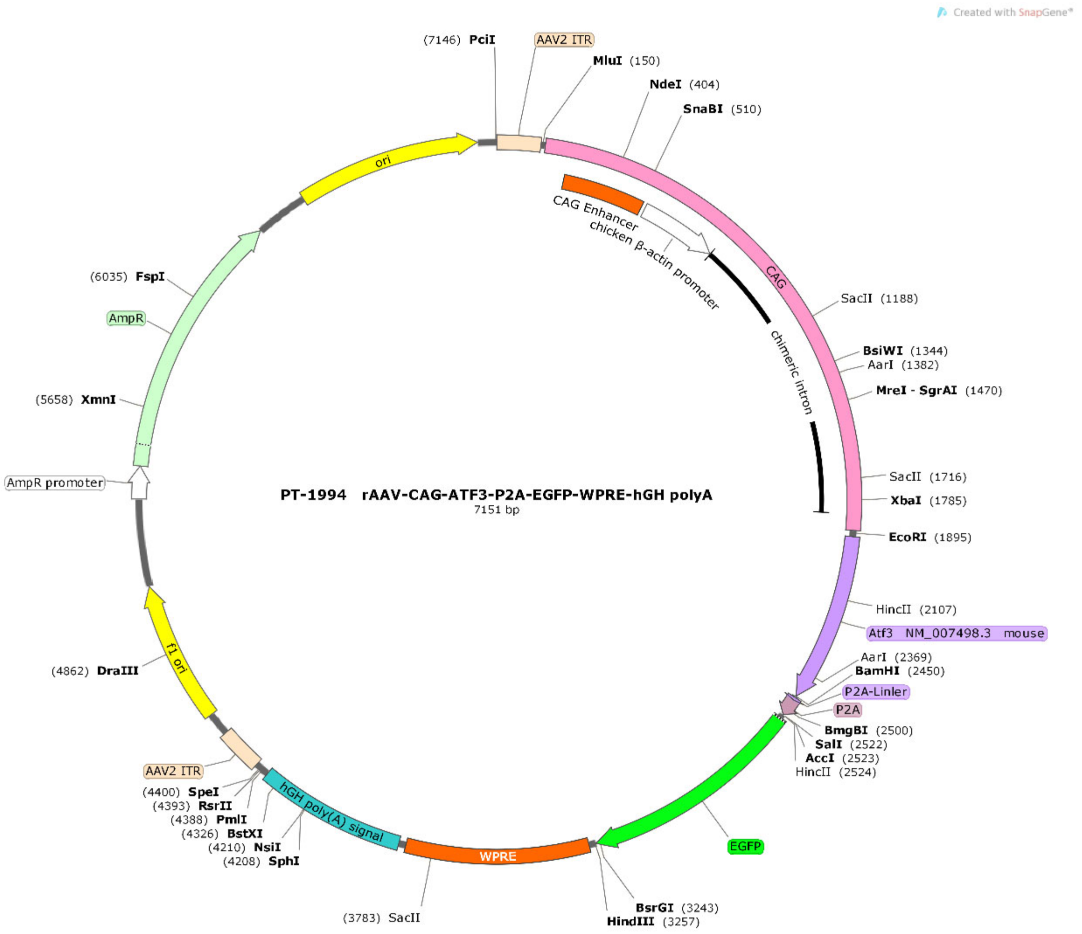

Supplementary Figure 2. Adeno-associated virus ATF3 overexpression vector model diagram.
